# Complete mitochondrial genome of the catophragmid barnacle *Catomerus polymerus* (Cirripedia, Thoracica, Balanomorpha, Catophragmidae)

**DOI:** 10.1080/23802359.2018.1532843

**Published:** 2018-10-26

**Authors:** Benny K. K. Chan, Lucia Aguilar, Bo Kyeng Hou, Hyun Mi Kang, Se-Joo Kim

**Affiliations:** a Biodiversity Research Center, Academica Sinica, Taipei, Taiwan;; b Institute for Conservation Biology & Environmental Management, Biological Sciences, University of Wollongong, NSW, Australia;; c Genome Editing Research Center, Korea Research Institute Bioscience and Biotechnology, Daejeon, Korea;; d Stem Cell Research Center, Korea Research Institute Bioscience and Biotechnology, Daejeon, Korea

**Keywords:** *Catomerus polymerus*, barnacle, mitochondrial genome, Catophragmidae, Chthamaloidea

## Abstract

The family Catophragmidae is one of the lower balanomorphs from traditional and recent multiple mitochondrial and nuclear markers molecular analysis. Here, we characterized the first mitogenome of the catophragmid barnacle *Catomerus polymerus*, which was 15,446 bp in length with a 68.3% AT content. The mitogenome had the typical pancrustacean gene arrangement, which was identical to the mitogenome configurations of the chthamalid *Octomeris* sp. and pachylasmatoid *Eochionelasmus ohtai*. On the mitogenomic tree, the catophragmid *Catomerus polymerus* formed an independent branch that was basal to the members of the superfamilies Tetraclitoidea and Balanoidea, which was inconsistent with previous findings.

Three superfamilies are recognized as the lower balanomorph barnacles from traditional barnacle systematics: Chionelasmatoidea, Chthamaloidea, and Pachylasmatoidea. These are characterized by six- to eight-shell plates or having small primodial plates the basal part of the external shell. A previous molecular phylogenetic analysis found that although the families Waikalasmatidae, Chionelasmatidae, Catophragmidae, Chthamalidae, and Pachylasmatidae were monophyletic with high bootstrap support, their relationships could not be resolved (Chan et al. 2017). As of 21 August 2018, GenBank contained four complete mitochondrial genomes (mitogenomes) of chthamalid and pachylasmatoid barnacles, but no mitogenomes of the other families. To understand their phylogenetic relationships, we determined the first mitogenome of the catophragmid barnacle *Catomerus polymerus* (Darwin).

In 14 January 2015, *C. polymerus* specimens were collected from Coledale Beach, Wollogong, Australia (34°17′S and 150°57′E). Genomic DNA extraction, sequencing, gene annotation, and phylogenetic analyses followed the methods of Kim et al. (2017, 2018). The specimen used for the mitogenome analysis has been deposited in the Biodiversity Research Museum of Academia Sinica, Taiwan (ASIZCR).

The complete mitogenome of *C*. *polymerus* was 15,446 bp in length (GenBank accession no. MH791045) and consisted of 13 protein-coding genes (PCGs), two ribosomal RNAs (rRNAs), 22 transfer RNAs (tRNAs), and a non-coding region. The gene organization followed the typical pancrustacean gene arrangement and was identical to that of the chthamalid *Octomeris* sp. (KJ754820) and pachylasmatoid *Eochionelasmus ohtai* (NC_036957). The tRNA arrangement differed slightly from that of the chthamalid *Notochthamalus scabrosus* (NC_022716), which possesses 21 tRNAs, while the chthamalid *Chthamalus antennatus* (NC_026730) had an unusual inverted gene arrangement between tRNA*^Phe^*and ND2.

The base composition was 33.7% A, 19.2% C, 12.4% G, and 34.6% T. All of the PCGs had an ATN start codon, except COX1, for which the start codon was not determined. Most of the PCGs terminated with a complete stop codon (TAA or TAG), although COX3, ND3, and ND4 had incomplete stop codons (T-). The 16S and 12S rRNAs were 1312 bp (73.6% AT content) and 749 bp (67.4% AT content), respectively. The tRNA genes ranged from 58 to 70 bp in size. A 298-bp-long (80.9% AT content) non-coding region was located between the 12S rRNA and tRNA*^Lys^*.

Phylogenetic trees were constructed with the PCGs of 15 barnacles using maximum likelihood and Bayesian inference (Figure 1). Previous studies based on nuclear and partial mitochondrial genes recognized that Catophragmidae shares a common ancestor with Waikalasmatidae, Chionelasmatidae, and Pachylasmatidae, although this lacked high bootstrap support or Bayesian posterior probability (Pérez-Losada et al. 2014; Chan et al. 2017). However, on the mitogenomic tree, the catophragmid *Catomerus polymerus* formed an independent branch and was located basal to the superfamilies Tetraclitoidea and Balanoidea. Therefore, further mitogenomic analysis of undetermined taxa of Waikalasmatidae and Pachylasmatidae is required to confirm the phylogenetic relationships and evolution of lower balanomorph barnacles.

**Figure 1.**
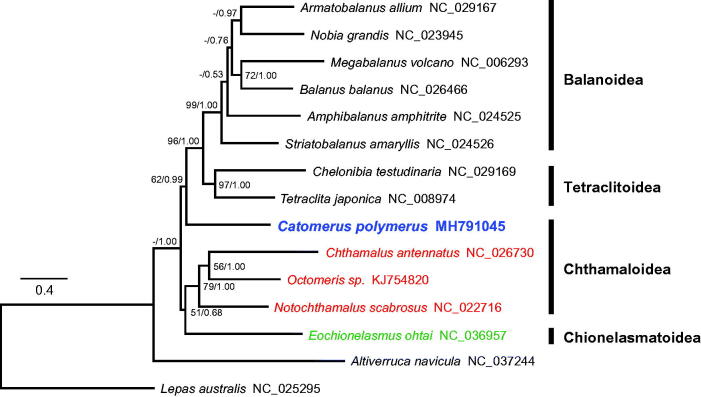
Phylogenetic tree of *Catomerus polymerus* and other thoracican barnacles based on 13 mitochondrial protein-coding genes. The model GTR + I + G was selected as the best evolutionary model using jModelTest 2.1.4. Numbers on internodes are the maximum likelihood bootstrap proportions (left) and Bayesian posterior probabilities (right). An asterisk indicates a bootstrap value of less than 50%.
